# Methods for assessment of the tumour microenvironment and immune interactions in non-small cell lung cancer. A narrative review

**DOI:** 10.3389/fonc.2023.1129195

**Published:** 2023-04-18

**Authors:** Kanishka Rangamuwa, Christian Aloe, Michael Christie, Marie-Liesse Asselin-Labat, Daniel Batey, Lou Irving, Thomas John, Steven Bozinovski, Tracy L. Leong, Daniel Steinfort

**Affiliations:** ^1^ Department of Respiratory Medicine, Royal Melbourne Hospital, Melbourne, VIC, Australia; ^2^ Department of Medicine Royal Melbourne Hospital (RMH), University of Melbourne, Parkville, VIC, Australia; ^3^ School of Health and Biomedical Sciences, RMIT University, Bundoora, VIC, Australia; ^4^ Department of Pathology, Royal Melbourne Hospital, Melbourne, VIC, Australia; ^5^ Personalised Oncology Division, Walter Eliza Hall Institute, Melbourne, VIC, Australia; ^6^ Peter MacCallum Cancer Centre, Melbourne, VIC, Australia; ^7^ Department of Respiratory Medicine, Austin Hospital, Heidelberg, VIC, Australia

**Keywords:** cytometry, immunohistochemistry, RNAseq analysis, non-smal cell lung cancer, tumour microenvironment, immune interaction, immunotherapy

## Abstract

Non-small cell lung cancer (NSCLC) is one of the leading causes of cancer death worldwide. Immunotherapy with immune checkpoint inhibitors (ICI) has significantly improved outcomes in some patients, however 80-85% of patients receiving immunotherapy develop primary resistance, manifesting as a lack of response to therapy. Of those that do have an initial response, disease progression may occur due to acquired resistance. The make-up of the tumour microenvironment (TME) and the interaction between tumour infiltrating immune cells and cancer cells can have a large impact on the response to immunotherapy. Robust assessment of the TME with accurate and reproducible methods is vital to understanding mechanisms of immunotherapy resistance. In this paper we will review the evidence of several methodologies to assess the TME, including multiplex immunohistochemistry, imaging mass cytometry, flow cytometry, mass cytometry and RNA sequencing.

## Introduction

Non-small cell lung cancer (NSCLC) accounts for over 80% of all lung cancer (1), which is the leading cause of cancer death worldwide (2). Median survival for those with advanced stage NSCLC is 12 months with conventional treatment of chemotherapy and radiotherapy (3). The advent of immune checkpoint inhibitor (ICI) therapy (immunotherapy) has significantly improved outcomes in some patients, however a durable response is seen in less than 30% of patients (4). Additionally, some patients that do have an initial response to immunotherapy can go on to develop resistance (5, 6).

Response to ICI therapy is related to several tumour, host and environmental factors (7). This includes intrinsic tumour properties such as cytokine release (7) and genetic composition (7) (including tumour mutational burden); and extrinsic factors such as the gut microbiome (8–13) and the presence of infection (7). These factors promote and suppress cancer immunity and sit in an equilibrium that is defined as the cancer-immune set point. This threshold needs to be surpassed for an individual with cancer to respond to ICI therapy (7).

Many studies, conducted in several different solid organ cancers, have shown that the make-up of the tumour microenvironment (TME) and the interaction between immune and cancer cells appears to have a large impact on response to ICIs and the ability to overcome the cancer immune setpoint. Assessment of histologic samples prior to initiation of therapy have demonstrated several immune phenotypes that predict response to immunotherapy. These profiles are immune-inflamed, immune-excluded and immune-desert phenotypes (14). The immune-inflamed phenotype is characterised by a TME where immune cells (especially CD4+ and CD8+ T cells) and cancer cells are in close proximity within the tumour parenchyma. These tumours are also associated with elevated levels of pro-inflammatory cytokines that promote T cell activation and expansion. The immune-excluded phenotype demonstrates abundant immune cells; however, these cells are found in the stroma and do not penetrate the tumour parenchyma. The immune-desert phenotype shows a paucity of T cells in both the stroma and parenchyma of the tumour (15, 16). In colorectal cancer the presence of specific T cell populations within the tumour microenvironment has been shown to correlate with survival (17) which has in turn led to the development of an immune score to aid with the staging of colorectal cancer (18, 19). Similar findings have been seen in lung cancer, where the density of tumour infiltrating lymphocytes in resectable adenocarcinoma has been associated with improved survival (20). Additionally, in lung cancer and melanoma, the presence of tumour infiltrating lymphocytes and increased gene expression for CD4 and CD8 was found to correlate with improved survival in those patients treated with immunotherapy (21).

Resistance to immunotherapy appears to be influenced by similar intrinsic and extrinsic factors. Tumour intrinsic factors include the tumour mutational burden, heterogeneity of tumour neoantigens, and expression of oncogene and tumour suppressor genes (14). Extrinsic factors influencing resistance are similarly strongly linked to the TME (14, 22) and the complex interactions between cancer cells and the immune system (23).

These observations demand that having robust, accurate and reproducible methods to assess the TME and immune interactions between NSCLC and the host immune system are vital to understanding mechanisms of immunotherapy resistance. From a practical standpoint, a majority of patients undergoing treatment with immunotherapy will have advanced stage NSCLC which are not amenable to surgical resection. As such, the majority of tissue samples acquired from patients with metastatic disease will be small volume and obtained through minimally invasive procedures such as bronchoscopy with endobronchial ultrasound (EBUS). This highlights the importance of validated methods to assess the TME and immune response in small volume samples and peripheral blood. Here we review key methods for assessing the tumour-immunity interaction in small volume specimens, and discuss potential limitations in their application in NSCLC (**Table 1**), as well as exploring the existing evidence base in lung cancer and other malignancies.

**Table 1:** Summary of techniques used to assess TME.

Methods		Number of markers	Throughput	Spatial Information	Precise quantification	Access to public data sets
IHC based	mIHC	4-9	Medium	Yes	Yes	No
	IMC	~40	Medium	Yes	Yes	No
	MIBI	~40	Medium	Yes	Yes	No
Cytometry based	Flow Cytometry	~20	Medium	No	Yes	No
	Mass Cytometry	~42	Medium	No	Yes	No
Transcriptomics	RNA sequencing (Bulk)	~hundreds	High	No	Yes	Yes
	Spatial RNA sequencing	~hundreds	Medium	Yes	No	Yes
	Single cell RNA sequencing	~hundreds	Medium	No	Yes	Yes

IHC, immunohistochemistry; mIHC, multiplex immunohistochemistry; IMC, Imaging mass cytometry; MIBI, Multiplex ion beam imaging.

## Immunohistochemistry-based methods

In lung cancer, immunohistochemistry (IHC) is routinely used in clinical practice to determine tumour type (24). It can also be used to assess the TME. For example, IHC is used to assess the expression of Programmed Death-Ligand 1 (PD-L1) (24) which is a marker of response to immune checkpoint inhibitors (25, 26), and to assess alteration in response to treatment (27). IHC provides information regarding types of cell populations, their characteristics, and the spatial relationship of different cell populations and tissue structures.

### Immunohistochemistry/immunofluorescence

IHC uses a primary antibody to target proteins of interest. Target proteins usually reflect the type and function of cell populations. A secondary antibody conjugated to an enzyme such as horseradish peroxidase is then applied before applying a chromogen substrate of the enzyme that will develop a colour upon enzymatic activity. This method enables amplification of the signal for the detection of the target protein with light microscopy (28). Additionally, IHC can be performed effectively on small tissue samples such as those obtained via EBUS (29).

Immunofluorescence (IF) uses a similar principle to chromagen-based IHC and can also be used to assess the TME. Here, instead of an antibody bound to an enzyme, antibodies are conjugated to fluorophores that can be detected by fluorescence microscopy (30). IF can either be performed directly where the primary antibody is attached to a fluorophore or indirectly where the fluorophore is attached to a secondary antibody which recognises the primary antibody of interest. Indirect IF allows for signal amplification (30).

While routinely used in clinical practice, IHC only allows for the assessment of a one or two proteins, and IF up to four, which limits comprehensive TME analysis. Multiplex immunohistochemistry (mIHC) has emerged as a tool for simultaneous detection of multiple biomarkers. However, there are several challenges to doing this accurately in a time-efficient manner. For example, performing co-staining with standard chromogen-based IHC is possible, however staining strategies are needed to overcome antibody cross-reactivity. Furthermore, due to overlap of chromogenic spectra, only up to 3 antibodies can be assessed this way. High-dimension multiplex IHC offers an alternative where antibodies and chromagen are stripped after each cycle of staining (31). This method enables staining of up to 15 proteins but is labour-intensive and cycles of staining/antibody stripping can cause tissue degradation (32). In addition, there is no robust analytical method to quantify cell populations with this approach (33). Similarly, while multiplex IF can assess up to 4 antibodies simultaneously, there are significant limitations. The most important considerations are spectral overlap of fluorophores and autofluorescence of the tissue (33).

Several methods such as tyramide signal amplification (OPAL™ mIHC), DNA barcode technology (CODEX ™, InSituPlex ™), image cyclic staining technology (MACSima ™), imaging mass cytometry (IMC) and Multiplex Ion Beam Imaging (MIBI) have been developed to provide more thorough assessment of the TME (33–38). DNA barcoded technologies and image cyclic staining technologies both have a paucity of published data (36). As a result, this paper will focus on OPAL™ mIHC, IMC, and MIBI. MIBI and IMC can be classified under mass cytometry-based imaging techniques as both use metal isotopes bound to antibodies. OPAL mIHC allows for simultaneous assessment of 4-9 antibodies (33, 39), while next generation IHC methods such as IMC and MIBI can assess up to 40 antibodies simultaneously. These techniques allow for assessment of both immune cell populations, their function and spatial arrangement in the TME (37, 38).

### OPAL multiplex immunohistochemistry (OPAL mIHC)

OPAL mIHC uses a tyramide signal amplification (TSA) system. This method uses a primary antibody to target the protein of interest and a secondary antibody that is conjugated to horse radish peroxidase. A tyramide-conjugated fluorophore is catalysed by horseradish peroxidase and will covalently bind to tyrosine residue in secondary antibody and tissue with the protein of interest. The primary and secondary antibodies can then be stripped, and the process repeated several times for different proteins of interest (33). OPAL mIHC allows for simultaneous assessment of up to 9 proteins in formalin-fixed and paraffin-embedded (FFPE) tissue samples (39).

Once stained slides are scanned, digital images are analysed using various software packages to obtain both spatial and quantitative information of the TME. Several software packages are available for analysis including commercial software such as HALO (Indica Labs, Albuquerque, New Mexico, USA) (40) and INFORM (Akoya Bioscience, Menlo Park, California, USA) (33), and open-source software such as QuPATH (41). Whole slide analysis is possible with mIHC. Analysis involves cell segmentation and annotation, cell phenotyping and spatial analysis. Development of machine learning models has allowed for the automation of some image analysis (42). Machine learning methods have also been developed to quantify immune cells in TME (43).

#### Clinical studies utilising OPAL mIHC in lung cancer

OPAL mIHC has been used to define the TME in several malignancies including lung cancer (44–48). Almost all studies assessed surgical resection samples (44–48), highlighting the need for studies validating the utility of this technique in small volume samples to evaluate the TME. Schalper et al. demonstrated that cytotoxic T-cell infiltration was associated with survival in NSCLC (47). mIHC has also shown that the spatial distribution of various T cell subclasses can impact survival in NSCLC (44, 49). It has also shown that neoadjuvant chemotherapy can increase PD-L1 expression and CD4+ T-cells in the TME (46). Other studies have shown that mIHC can be used to assess functional cellular molecules such as PD-L1, indoleamine 2,3-dioxygenase 1 (IDO-1) and B7-H4 and their significance in NSCLC (48).

### Mass cytometry-based imaging

Like mIHC, mass cytometry-based imaging techniques have also been used to define the TME. These techniques utilise target antibodies attached to metal isotopes to assess multiple target proteins simultaneously. Two such techniques are MIBI and IMC.

MIBI uses secondary mass spectrometry to image primary antibodies that are coupled with rare metal isotopes. Firstly, FFPE sections are stained with rare earth metals conjugated primary antibodies. These are then loaded on to the MIBIscope and rasterized with a xenon duoplasmatron primary ion beam. This results in the release of secondary ions which are subsequently analysed with a magnetic sector mass spectrometer with time-of-flight (TOF) analysis. Multiple isotopes can be assessed simultaneously with most devices analysing up to 40 isotopes at a time. The resultant data provides a two-dimensional map of the distribution of each isotope and its corresponding antibody and its related epitope (37). Analysis of MIBI images can then be performed via computational pipelines that allow for automation of most steps (42, 50).

Like MIBI, IMC utilises rare earth metal labelled antibodies to stain FFPE specimens. Areas of interest within these specimens are then ablated by a laser, and the resulting tissue plumes are assessed by time-of-flight cytometry to determine isotope abundance (38, 51). IMC can analyse a similar number of antibodies as MIBI (52), however repeat analysis is not possible on the same section of tissue due to complete ablation (53). Mass cytometry-based imaging has considerable advantages over mIHC. Autofluorescence and spectral overlap are not a concern with mass-cytometry imaging and staining of the section in one single step ensures tissue integrity compared to mIHC where cyclic staining can lead to tissue degradation (37).

#### Clinical studies utilising MIBI and IMC in lung cancer

Keren et al. have described a robust workflow for antibody labelling, image acquisition and analysis pathways for assessing cell populations, functional capacity, and the spatial relation of cells to each other in triple negative breast cancer, which is applicable to other tissue types (50, 54).

The TME in breast cancer has been assessed extensively with MIBI. Studies have demonstrated the importance of programmed cell death protein-1 (PD-1), PD-L1, indoleamine 2,3-dioxygenase (IDO) and lymphocyte activation gene 3 (LAG-3) in cellular interactions, linking these immunoregulatory proteins to recurrence and survival (55). Other studies have shown that disruption of myoepithelial cells in ductal carcinoma *in situ* (DCIS) can reduce the risk of progression to invasive breast cancer (56). The workflow demonstrated in these studies appears generalizable to other cancer types. With regards to lung cancer, MIBI has not been used extensively to define the TME, however Ptacek et al. demonstrated the feasibility of using MIBI to assess TME in NSCLC (57). In this study MIBI was used to simultaneously defined multiple cell types including, T cells, B cells and macrophages in spatial relation to tumour cells. Additionally, McCaffrey et al. used MIBI to evaluate the immunoregulatory microenvironment of *Mycobacterium tuberculosis* granulomata (58) using a combination of lung biopsy samples and resection samples (58).

While assessment of TME using the above methods is robust, the applicability in patients with NSCLC on immunotherapy is still unclear. Many patients with resistance to immunotherapy would undergo percutaneous or bronchoscopic biopsy of the cancer in question and further work to validate these multiplex techniques and their validity in small biopsy samples is needed.

## Cytometry based techniques

Cytometry techniques provide single cell analysis of tumour tissue and can also be used to assess the TME (59, 60). Flow cytometry (FACS) and mass cytometry (CyTOF) are two commonly used methods of cytometry that we will review here. Both methods utilise a single cell suspension, meaning information about spatial relationships between cells is lost (38). The need to create cell suspension may lead to cell type loss and limits analysis to fresh tissue, meaning FFPE samples from historic data sets cannot be assessed with this method (59).

### Flow *cytometry*

Flow cytometry uses fluorophore-conjugated antibodies to target molecules of interest. A suspension of cells bound to antibodies is exposed to a laser, one cell at a time. This causes fluorophore excitation which leads to emission of light at a particular wavelength that can be detected by a sensor. Multiple antibodies can be assessed simultaneously (up to 24 with some devices that offer spectral unmixing) (60). Flow cytometry allows for assessment of thousands of events per second (60). It can assess large numbers of cells quickly and provide extensive data with regards to cell populations and their function (60). The main limitations of flow cytometry are the limited number antibodies that can be assessed at one time and the overlap in emission spectra of some fluorophores meaning that antibody combinations need to be selected carefully (60).

#### Clinical studies utilising flow cytometry in lung cancer

Studies in NSCLC have used flow cytometry to define populations of monocytes (61), dendritic cells (62) and lymphocytes (63) in peripheral blood. For example, HLA-DR^low^ monocyte populations in peripheral blood correlate well with high neutrophil/lymphocyte ratio (NLR) and have a negative association with survival (61). Another study has shown that patients with NSCLC have significantly lower levels of peripheral blood plasmacytoid dendritic cells (pDC), this was again lower in patients with metastatic disease than those with early-stage NSCLC suggesting some prognostic value to pDCs (62). Defining lymphocyte populations in blood has important implications as well, for example increased proportions of regulatory T cells in peripheral blood was seen in patients with NSCLC who had progressive disease despite radiotherapy (63). Similarly elevated levels of circulating monocytic myeloid-derived suppressive-like cells observed with flow cytometry have been shown to be associated with resistance to immune checkpoint inhibitor therapy (64).

Flow cytometry has also been used to define the TME and its immune cells in NSCLC (65). For example, Bonnal et al. used flow cytometry in conjunction with single cell RNA sequencing to show that eomesodermin homolog (*EOMES*)^+^ type 1 regulatory T-like cells are associated with disease progression in NSCLC (66). Additionally, flow cytometry and IHC have been used to define tumour-associated macrophages in NSCLC (67) and that regulatory T cells are seen in higher numbers in NSCLC and is associated with reduced natural killer (NK) cells when compared to tumour-free lung tissue (68). While flow cytometry does provide robust assessment of cell populations and function, most studies in humans have been performed on resection specimens as opposed to biopsy specimens.

### Mass *cytometry* (CyTOF)

Mass cytometry uses antibodies conjugated to heavy metal isotopes to target the molecules of interest. Once the cell suspension has been labelled with these antibodies, it is introduced into a mass spectrometer where an argon plasma creates an ionised cloud of atoms that is enriched for heavy metals. The heavy metal ions are then separated by time-of-flight mass spectrometer by their mass to charge ratio. This is then detected as an electrical signal at the terminal gate, the ion count reflecting the expression of the corresponding target molecule. Mass cytometry allows for the simultaneous assessment of up to 40 antibodies (59). Mass cytometry has been validated against flow cytometry for assessment of both peripheral blood mononuclear cells (PBMC) and tumour tissue in human cancer studies (69). Mass cytometry provided similar analysis to flow cytometry with respect to cell populations, function, activation, and exhaustion. Fewer cell numbers are also needed for analysis when compared to traditional flow cytometry (69).

#### Clinical studies utilising mass cytometry in lung cancer

Similar to flow cytometry, mass cytometry has been used to define PBMC populations in studies of NSCLC (70). For example, CD33 expression on PBMC monocytes has been shown to predict response to anti-PD-1 immunotherapy in NSCLC (71). Mass cytometry has been used for the assessment of the TME in NSCLC where CyTOF was used in conjunction with multiplexed IF to define the significance of PD-1, LAG-3 and T cell immunoglobulin and mucin domain-containing protein 3 (TIM-3) expression in tumour infiltrating lymphocytes (72). Mass cytometry has also been used to correlate NLR, CD9 and HLA-DR expression with the prevalence of tertiary lymphoid structures in patients with NSCLC (73). In these studies, resected samples of NSCLC were used for analysis with mass cytometry (72).

Both flow cytometry and mass cytometry can play a key role in defining immune cell populations and their function in both the TME and peripheral blood. To our knowledge, nearly all studies that have used cytometry to assess the TME have utilised resected tissue samples. Weeden et al. used tissue samples obtained with EBUS for CyTOF analysis. Robust data were obtained from the analysis of EBUS samples from 12 patients with advanced-stage lung cancer with CyTOF (74). However, as mentioned for the previous techniques described above, further validation of these methods with small biopsy samples would improve the applicability of defining TME in patients that develop resistance to immunotherapy.

## Transcriptomics and gene expression

RNA sequencing is another powerful tool that has been used to assess oncological disease at a molecular level (75, 76). Next generation RNA sequencing (RNA-seq) techniques that allow for high throughput sequencing has been available since 2005 (77), and has shown to be able to quantify tumour infiltrating immune cells using bioinformatic pipelines (78). The accuracy and reproducibility of RNA-seq depends on the specific platform, though in a broad sense RNA-seq allows for accurate, fast high throughput sequencing at low cost (79). RNA sequencing in general refers to bulk RNA sequencing, where tissue is sequenced without sorting into single cells. In comparison, single cell RNA sequencing (scRNAseq) which was developed in 2009, allows for assessment of the transcriptome at a single cell resolution (80). More recently spatial transcriptomic methods provide additional data regarding the spatial orientation of various RNA signatures (81).

### RNA *sequencing* (Bulk RNA sequencing)

RNA-seq techniques can vary depending on the platform that is used; however, most platforms involve RNA extracted from cells of interest, followed by RNA fragmentation. Improvements in library preparation techniques has allowed for improved assessment of degraded tissue, including FFPE blocks. Reverse transcription is then performed on RNA fragments to obtain complementary DNA (cDNA) which then undergoes PCR amplification to create sequencing libraries. A DNA polymerase is then used to sequence the libraries (82).

There are many pipelines available for computational analysis once sequencing is completed. While the specific type of analysis will depend on the purpose of the experiment, most analysis will follow the following broad steps. Quality control is first performed to determine the quality of sequencing and eliminates low quality reads. This is followed by read alignment where clean RNA reads are mapped to a human reference transcriptome. Once aligned, the short reads are assembled into transcripts. The expression of the transcript can then be quantified. This will routinely generate an expression matrix that can then undergo further statistical analysis (82).

Several bioinformatic analysis techniques have been developed to determine TME composition and include deconvolution techniques (83), gene set enrichment analysis (84) and other techniques that can quantify immune populations such as xCell (85). Deconvolution methods such as CIBERSORT (83) estimate cell populations based on overall signature (83) while gene set enrichment analysis determines differences in defined gene sets and can assess different gene signatures for immune cells (84). Whereas xCell uses a gene signature to map to 64 corresponding stromal and immune cells (85). These analysis techniques can be applied to large data sets, including publicly available data sets such as The Cancer Genome Atlas (TCGA) data base or datasets deposited on the Gene Expression Omnibus (GEO).

### Single-cell RNA sequencing

In addition to bulk RNA sequencing, single-cell RNA sequencing can profile the transcriptome at a single cell resolution (86). Analysis of different gene expression between individual cells can potentially identify rare populations that would not be detected with pooled analysis. This can be particularly useful for determining heterogeneity in immune cell populations in the TME (82).

Single-cell RNA sequencing first requires single cell isolation, which can be done by different methods including, limiting dilution (87, 88), micromanipulation (87, 88), flow activated cell sorting (89), laser capture microdissection (90), and microfluidic technology (91). Once single cell isolation has been performed, cells are lysed, and RNA then undergoes reverse transcription to cDNA. This is then followed by amplification and sequencing. While there are pros and cons of each technique, this will not be addressed here. Overall, when compared to bulk RNA sequencing more cells are required due to the loss of cells at time of isolation, lysis and mRNA capture (86, 92).

Analysis pipelines for single-cell RNA sequencing differs from bulk RNA sequencing as there is increased noise as well as technical and biological variability. Once quality control and normalisation have been implemented, analysis can be performed at a cellular level to cluster and classify cell types. Gene level analysis can then determine differences in differentially expressed genes between clusters to infer differences in function between cell clusters (86, 92). With both bulk RNA sequencing and single cell RNA sequencing, spatial information that would be available with IHC methods is absent. Spatial transcriptomics, which was first described in 2016 can be used to overcome this limitation (81).

### Spatial transcriptomics

Spatial transcriptomics uses positional barcodes to map cDNA to particular areas of tissue, which will then demonstrate variations in gene expression with respect to spatial orientation (81). To our knowledge spatial transcriptomic methods have not been performed on small biopsy samples.

#### Clinical studies utilising transcriptomic methods in lung cancer

RNA-seq has been used in many studies to define changes in the TME in order to quantify tumour-infiltrating immune cells and possibly predict the response to immunotherapy. For example, Casarrubois et al. used RNA-seq to classify the TME in patients with NSCLC undergoing neoadjuvant chemo-immunotherapy. This study showed that patients with complete pathologic response had a higher baseline immune infiltrate characterised by higher levels of interferon gamma (*IFNG)*, granzyme B *(GZMB)*, natural killer cell granule protein 7 (*NKG7)* and M1 macrophages (93). Additionally several studies have validated the use of RNA-seq using small volume samples including endobronchial ultrasound-guided transbronchial needle aspirate (EBUS-TBNA) (94) and cytology samples from bronchoscopic transbronchial brushing specimens (95–97). For example, RNA-seq can be performed on EBUS samples to determine PD-L1 expression. This has been shown to correlate well with PD-L1 expression via IHC, which is currently used to predict response to immunotherapy (98).

Similarly, RNA-seq has been used to assess predictors of resistance to immunotherapy. Analysis of 624 patients with squamous cell lung cancer from the TCGA and GEO data sets identified an immune exhausted subclass of tumours, classified by an upregulation of inhibitory checkpoints, M2 macrophages and CD4+ regulatory T cells. These tumours were associated with poor prognosis and deemed to be associated with resistance to immunotherapy as predicted by tumour immune dysfunction and exclusion algorithm (99). Another study assessed immune escape mechanisms in 48 patients with NSCLC treated with ICI and showed alterations in antigen processing and PD-L1 expression played a key role in immune escape in this cohort of patients (100).

Single-cell RNA sequencing has also been used to assess TME in NSCLC. For example, Bischoff et al. demonstrated two distinct patterns of TME in patients with lung adenocarcinoma where an Immune-activated TME characterised by pro-inflammatory monocyte-derived macrophages, NK cells, pDCs and exhausted CD8+ T cells was shown to have a poor prognosis (101). Additionally, Zhang et al. used spatial transcriptomics to show that TME in brain metastasis from NSCLC is characterised by reduced antigen presenting, B/T cell function and increased neutrophils and M2 macrophages (102).

## Summary

Assessment of the TME will play a vital role in further understanding the mechanisms of immune escape and resistance to immunotherapy in patients with NSCLC. The techniques discussed in this paper have the potential as both standalone techniques or in combination to play a key role in this, as we push towards a more personalised approach to lung cancer care. As described, most of the techniques described have either been used in other cancers, blood samples or resection samples. However, as most patients eligible for immunotherapy have advanced disease, obtaining resection samples is not practical. Given this, implementation, and validation of these techniques to assess TME with small biopsy samples such as those obtained *via* bronchoscopy will be important in expanding future understanding of the tumour-immune interaction.

Immunostaining and cytometry-based methods will need to be better validated for small volume samples to provide meaningful insight into the TME, given the heterogeneity noted with TME. RNA sequencing-based techniques currently have the largest body of evidence, though further validation with spatial transcriptomic techniques would further add value to this approach. Utilisation of these techniques together will most likely have the most benefit.

## Author contributions

KR - Performed literature review and prepared manuscript. KR, DS, and TL - Conceived manuscript idea. DS and TL - co-supervisors. All authors - reviewed/edited manuscript. All authors contributed to the article and approved the submitted version.
